# Complete Genome Sequences of *Lactobacillus* Strains C25 and P38, Isolated from Chicken Cecum

**DOI:** 10.1128/MRA.00501-20

**Published:** 2020-09-24

**Authors:** Hosni M. Hassan, Mary Mendoza, Morvarid Rezvani, Matthew D. Koci, Allison N. Dickey, Elizabeth H. Scholl

**Affiliations:** a Prestage Department of Poultry Science, North Carolina State University, Raleigh, North Carolina, USA; b Bioinformatics Research Center, North Carolina State University, Raleigh, North Carolina, USA; University of Maryland School of Medicine

## Abstract

We report the complete circular genome sequences of Lactobacillus crispatus strain C25, its plasmid, and Lactobacillus animalis strain P38; both strains were isolated from the cecum of 4-week-old chickens. These isolates represent potential probiotic strains for poultry.

## ANNOUNCEMENT

The genus *Lactobacillus* is one of the largest in the lactic acid bacterial group (https://www.ncbi.nlm.nih.gov/genomes/lproks.cgi). Lactobacilli are found in diverse environments (1).

Previously, we published draft genomes of Lactobacillus crispatus strain C25 and Lactobacillus animalis strain P38, isolated from chickens (2, 3). Strain C25 was isolated from pooled cecum samples from seven 4-week-old female Leghorn chickens grown on a commercial diet; strain P38 was also isolated from pooled cecum samples and from chickens grown under the same conditions as C25, except that the diet contained galactooligosaccharide as a prebiotic (4). The draft genomes were assembled from Illumina MiSeq reads (2, 3). In this report, we resequenced strains C25 and P38 using PacBio to generate single-chromosome contigs. The original isolates were stored at −80°C in individual vials and never subcultured. Frozen cells were cultured in MRS medium and grown anaerobically for 20 h (2, 3), and the DNA was extracted using the Promega Wizard genomic DNA purification kit. The DNA samples were sheared using a Covaris g-TUBE, and size selection was performed using BluePippin. We used the SMRTbell template prep kit v1.0 to make the sequencing libraries with a target insert size of 20 kb. The sequencing primer was annealed to the libraries and the polymerase was bound using the Sequel binding kit v2.1. The bound complexes were loaded onto the Sequel system and sequenced with the Sequel sequencing kit v2.1 using 10-hour movie times. Quality filtering was done on the instrument automatically, and the subread .BAM files contained the high-quality filtered reads (Table 1).

**TABLE 1:** Sequencing metadata and accession numbers

Strain	SRA accession no.	GenBank accession no.	Avg insert size (kb)	No. of subreads	Subread *N*_50_ (bp)
C25	SRR10747684	CP047142, CP047143	23	1,138,538	7,684
P38	SRR10747683	CP047141	10	3,009,617	4,066

Each strain had 1 single-molecule real-time (SMRT) cell, and the HGAP4 pipeline from SMRT Link v5.1.0 was used for the genome assemblies (5). The default pipeline parameters were used, except for genome length (C25, 2.15 Mb; P38, 2.0 Mb) and seed coverage (C25, 40×; P38, 30×). The preliminary assemblies for C25 and P38 showed high coverage (>1,000×). To reduce the number of reads used in generating the assemblies, a subsample of the reads was selected with “samtools view –s” (seed = 0; sampling fractions, 0.1 [C25] and 0.053 [P38]). The C25 assembly had 2 contigs (mean coverage, 200×), and the P38 assembly had 1 contig (mean coverage, 233×).

Circlator was used for contig circularization (6); the C25 and P38 chromosomes were circular, and Circlator set their origin locations to *dnaA*. Strain C25 also had one circular plasmid, and Circlator set its origin to the gene closest to the center of the Circlator contig. The circularized assemblies were polished with the subsampled reads using Arrow from the GenomicConsensus package v2.2.2 in SMRT Link. The C25 chromosome and plasmid lengths are 2,145,933 bp (GC content, 37.0%) and 179,151 bp (GC content, 35.0%), respectively. The length of the P38 chromosome is 1,906,794 bp (GC content, 41.2%). The NCBI PGAP v4.10 was used for assembly annotation (7, 8). The total number of genes for C25 is 2,312 and for P38 is 1,884.

### Data availability.

The assemblies were deposited in GenBank under the accession numbers CP047142 and CP047143 for the C25 chromosome and its plasmid, respectively, and CP047141 for the P38 chromosome. The PacBio sequencing reads were deposited in the Sequence Read Archive (SRA) under the accession numbers SRR10747684 for C25 and SRR10747683 for P38.
